# Distribution, Size, and Shape of Abdominal Aortic Calcified Deposits and Their Relationship to Mortality in Postmenopausal Women

**DOI:** 10.1155/2012/459286

**Published:** 2012-06-06

**Authors:** Melanie Ganz, Marleen de Bruijne, Erik B. Dam, Paola Pettersen, Morten A. Karsdal, Claus Christiansen, Mads Nielsen

**Affiliations:** ^1^Department of Computer Science, University of Copenhagen, 2100 Copenhagen, Denmark; ^2^Nordic Bioscience Imaging A/S, 2730 Herlev, Denmark; ^3^Biomedical Imaging Group Rotterdam, Departments of Radiology & Medical Informatics, Erasmus MC, 3000 CA Rotterdam, The Netherlands; ^4^CCBR-Synarc A/S, 2750 Ballerup, Denmark; ^5^Nordic Bioscience A/S, 2730 Herlev, Denmark

## Abstract

Abdominal aortic calcifications (AACs) correlate strongly with coronary artery calcifications and can be predictors of cardiovascular mortality. We investigated whether size, shape, and distribution of AACs are related to mortality and how such prognostic markers perform compared to the state-of-the-art AC24 marker introduced by Kauppila. Methods. For 308 postmenopausal women, we quantified the number of AAC and the percentage of the abdominal aorta that the lesions occupied in terms of their area, simulated plaque area, thickness, wall coverage, and length. We analysed inter-/intraobserver reproducibility and predictive ability of mortality after 8-9 years via Cox regression leading to hazard ratios (HRs). *Results*. The coefficient of variation was below 25% for all markers. The strongest individual predictors were the number of calcifications (HR = 2.4) and the simulated area percentage (HR = 2.96) of a calcified plaque, and, unlike AC24 (HR = 1.66), they allowed mortality prediction also after adjusting for traditional risk factors. In a combined Cox regression model, the strongest complementary predictors were the number of calcifications (HR = 2.76) and the area percentage (HR = −3.84). *Conclusion*. Morphometric markers of AAC quantified from radiographs may be a useful tool for screening and monitoring risk of CVD mortality.

## 1. Introduction

Cardiovascular diseases (CVDs) are the prevalent cause of death in Europe [1] and the United States [2]. This is despite general acceptance that a healthy lifestyle and risk factor management can prevent the development of CVD [3]. Furthermore, two-thirds of women who die suddenly from CVD have no previously recognized symptoms [3]. Thus, it is essential to find effective and broadly applicable indicators of cardiovascular risk that may prompt timely intervention.

Current noninvasive modalities for imaging atherosclerosis are radiographs, ultrasound, computed tomography (CT), and magnetic resonance imaging (MRI) [4]. Ultrasound is used to visualize the carotid intima-media thickness (IMT) because carotid IMT has been shown to be associated with atherosclerosis [5] and is thus a marker for CVD. Multislice CT is able to quantify the degree of coronary artery calcification (CAC) with good reproducibility [6, 7], which provides a strong measure of cardiovascular risk [8] independently from, and potentially more powerful than, traditional risk factors such as smoking [9]. However, due to the relatively large exposure to ionizing radiation, use of clinical dose CT is not advisable in large-scale screening, but only to aid interventional treatment of patients at intermediate risk [10]. Low-dose CT, on the contrary, could be used to evaluate coronary calcifications for screening purposes [11], and only its cost is a limiting factor. MRI is a noninvasive modality to assess atherosclerosis in different vascular beds. However, MRI measurements are challenged by the size of the smaller arteries, and especially assessment of the coronary arteries is difficult due to cardiac and respiratory motion artefacts. Furthermore, also MRI still has to prove its cost-effectiveness for screening purposes.

An alternative to examining coronary arteries for calcification is to assess the abdominal aorta, since it is contrary to the coronary arteries accessible through radiographs. Abdominal aortic calcifications (AACs) are strong predictors of cardiovascular morbidity and mortality [12], correlate strongly with coronary artery calcifications, and may hence predict the risk of coronary artery problems [13, 14]. The state of the art methodology to estimate CVD risk from lumbar aortic radiographs is the abdominal aortic calcification score (AC24) proposed by the Framingham study group [15]. A big advantage is that such a AAC scoring can, for example, in the case of postmenopausal women, be performed without additional ionising radiation exposure or cost as these images are readily available from osteoporosis screening [16, 17].

We investigated if the morphometric aspects of the information that can be made available from CT, MRI, or ultrasound as described above could also be obtained from novel markers of AAC quantified from plain radiographs. Due to the semiquantitative grading of the AC24 score, such markers could potentially be more sensitive—in particular with respect to investigating the potential significance of smaller calcifications. For this, we outlined the boundaries of the calcified deposits in the lumbar aortic region and quantified the number of calcified deposits as well as the percentage of the abdominal aorta covered by calcifications in terms of area, simulated-plaque area, thickness, wall coverage, and length. These potential AAC markers were evaluated for precision and their ability to predict CVD-related mortality.

## 2. Materials and Methods

### 2.1. Study Population

308 females were selected from those who took part in the multicentre PERF study [18] who were examined radiologically in 1992, and examined again in 2001 in the followup EPI study [19]. We chose those whose interval between their first and second clinic visit was 8-9 years, with known alive/mortality status, who were postmenopausal, and whose lumbar aorta was visible on a single radiograph at baseline and at followup. Information about the mortality status was obtained via the Central Registry of the Danish Ministry of Health, and the death causes were grouped into three groups: CVD, cancer, and other causes. The studies were approved by the local ethics committee, and the patients signed informed consent forms.

### 2.2. Metabolic and Physical Measurements

At baseline, demographic information and CVD risk parameters such as age, weight, height, body mass index (BMI), waist and hip circumferences, systolic and diastolic blood pressure (BP), treated hypertension, treated diabetes, smoking, regular alcohol and daily coffee consumption, and weekly fitness activity were collected. Using a blood analyzer (Cobas Mira Plus, Roche Diagnostics Systems, Hoffman-La Roche, Basel, Switzerland), measurements of fasting glucose and lipid profile (total cholesterol, triglycerides, LDL-cholesterol (LDL-C), HDL-cholesterol (HDL-C), and apolipoprotein (ApoA and ApoB)) were obtained.

On the basis of these measurements, the composite risk markers, systemic coronary risk evaluation (SCORE) [20] and Framingham score [21], were calculated. The SCORE is a combination of the age, smoking status, levels of total cholesterol, and systolic blood pressure, while the Framingham score is comprised of the same variables plus the HDL-C and the hypertension treatment status.

### 2.3. Radiographic Analysis

**Figure 1:**
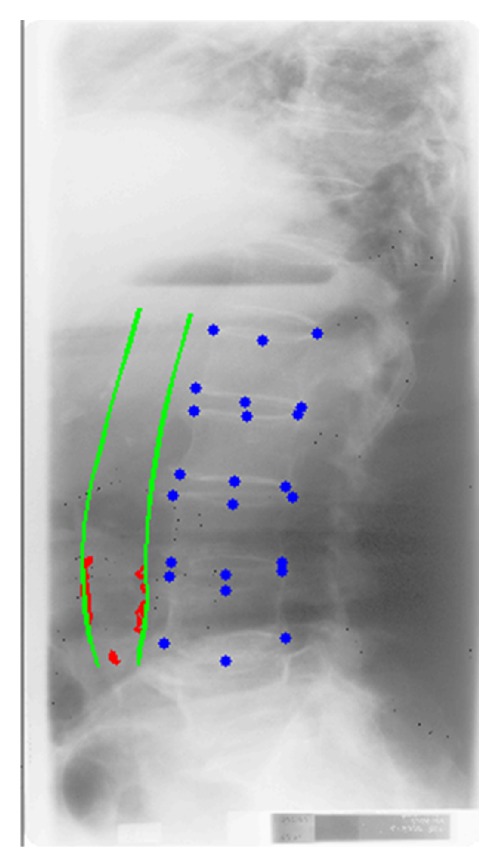
A manual annotation of an X-ray: in blue we see distinct vertebra points, in green the aorta wall, and in red the calcifications.

The lateral X-ray images of the lumbar aorta (L1-L4) were acquired on film in 1992 and 2001 [18, 19], respectively, and digitized in 2007/2008 using a DosimetryProAdvantage scanner (Vidar, Herndon, USA), providing an image resolution of 9651 × 4008 pixels on a 12-bit gray scale with a pixel size of 44.6 *μ*m × 44.6 *μ*m. Three trained radiologists without prior knowledge of the patients' conditions annotated the corner and mid points of the vertebrae (L1-L4), the corresponding abdominal aorta walls, and their calcifications in the digitized images manually. The three radiologists had ten, eight, and five years of experience. They used radiological reading units (Sectra, Linköping, Sweden) and annotation software specifically implemented for that task in Matlab (The MathWorks, Natick, USA), which allowed them to change brightness and contrast, zoom in and out, and to edit outlines, as seen in Figure 1.

**Figure 2:**
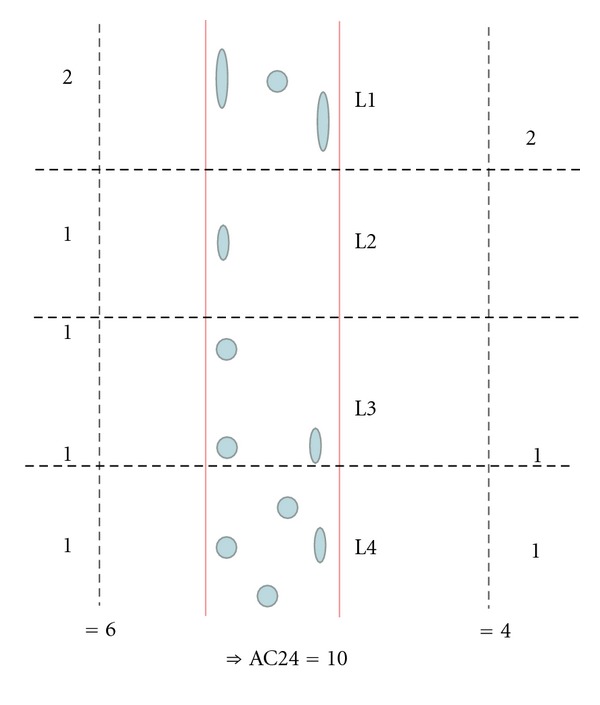
A schematic view of AC24. The AC24 is constructed by projecting the AAC to the corresponding aorta wall.

The AC24 [15] was constructed by projecting the AACs to the corresponding aorta wall. Then, the aortic sections adjacent to each vertebra L1-L4 were graded by the degree of lesion occupation: 0 for no AAC, 1 for AACs occupying less than 1/3 of the wall they were projected onto, 2 for AACs occupying more than 1/3, but less than 2/3 in the projection, and 3 for a 2/3 or more occupation of the wall. An example of an AC24 scoring can be seen in Figure 2. In addition to the AC24 scores provided by the radiologists, the outlines of the calcifications were used in an alternative computer-based computation of the AC24.

For all the images with calcifications, annotations were performed by one of the three different radiologists. For a subset of 8 images, annotations by two radiologists were made twice in order to evaluate inter- and intraobserver precision. Reoutlining was performed blinded to earlier outlines and separated by approximately six to eight weeks.

### 2.4. AAC Markers

The proposed AAC markers were automatically computed from the radiologist's computer-assisted outlines of calcified deposits in the radiographs.

**Figure 3:**
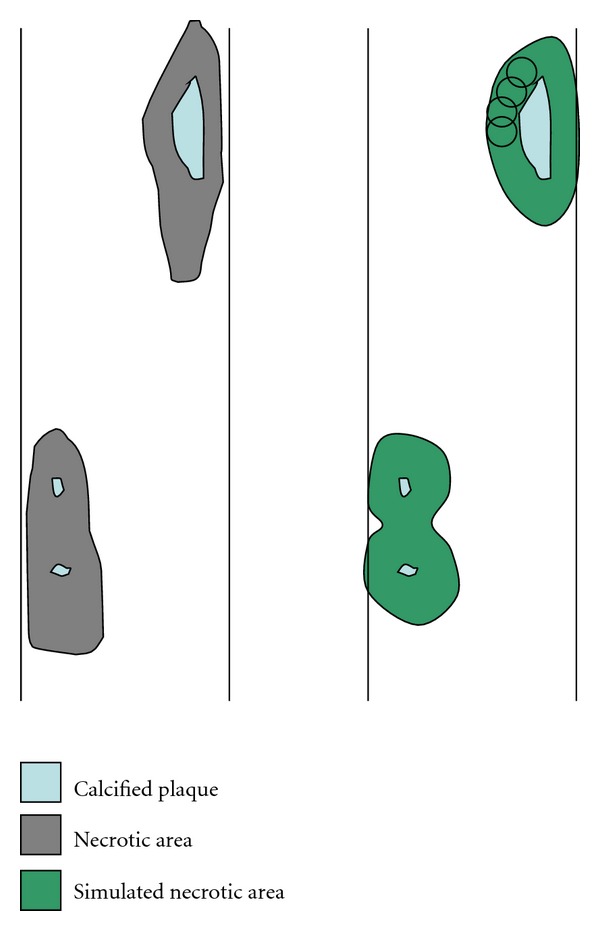
Left: a schematic visualization of a plaque similar to what can be seen in histology. The calcified plaque (light blue) is surrounded by an area of necrotic tissue (gray). Right: the simulated area tries to imitate the area of necrotic tissue (green) as seen in histology by a morphological dilation (visualized by circles) of the calcified plaque (light blue).

- Area percentage: the percentage of the area of the lumbar aorta adjacent to L1-L4 occupied by AACs.
- Simulated area percentage: we tried to estimate the size of the underlying atherosclerotic inflammation from the area and shape of the observed AACs since X-ray analysis can only visualize the calcified core of the AACs. The extent of the atherosclerotic inflammation was simulated by a morphological dilation [22] with a circular structuring element of radius 200 pixels (approximately 8.9 mm). The size of the structuring element was derived by a parameter study on a subset of the data, and it was confirmed to be biologically sensible by comparing with histology and image analysis observations which estimated the size of the atherosclerotic inflammation surrounding the calcified plaque to be between 3 mm [23] and 5–10 mm [24]. An illustration of this computer-based simulation of the full plaque area is given in Figure 3. The simulated area percentage is the percentage of the lumbar aorta covered by the simulated plaques including both calcified core and simulated inflamed area.

(iii) Thickness percentage: the average thickness of the AACs along the aorta wall relative to the aorta width.
(iv) Wall percentage: the percentage of the anterior and posterior lumbar aorta wall covered by AACs.
(v) Length percentage: the fraction of the length of the aorta where AACs were present at any position (anterior, posterior, or internal).
(vi) Number of calcified deposits: the number of distinct AACs visible between L1 and L4 in each radiograph.

We examined the degree to which these markers could be reliably established on the basis of manual annotations of X-ray images and evaluated their association to mortality, also when adjusted for metabolic or physical markers.

### 2.5. Statistical Analysis

Kendall's coefficient of concordance [25] was used to assess the level of agreement between AC24 scorings of calcified images made by radiologists directly on the original X-rays and AC24 scorings by the computer, based on the radiologist's annotation outlines.

To measure the inter- and intraobserver variability of the manual annotations of the radiologists on the 8 images allocated specifically for this purpose, we used the Jaccard Index (*A*) [26]. We computed the ratio of the area identified as calcified in two outlines, divided by the area identified as calcified in at least one outline:

(1) $$A=\frac{\left|A_{1}\cap A_{2}\right|}{\left|A_{1}\cup A_{2}\right|},$$

where *A*_1_ and *A*_2_ are a binary annotations. The Jaccard Index varies from 0 for no agreement to 1 for complete agreement. Typically, Cohen's *κ* [27] would be used to measure the inter-rater agreement for categorical items like pixels. However, the statistics will be dominated by the very large class of non-calcified pixels, and individual pixel scorings cannot be considered statistically independent.

 The inter- and intraobserver variability of the AAC markers computed from the radiologist's outlines was analysed on the 8 images by the mean coefficients of variation (CV).

The predictive power of mortality in terms of hazard ratio per standard deviation change (HR) of the individual AAC scorings was analysed by Cox regression [28, 29], where time of death was the outcome variable and survivors were right-censored. This analysis was performed on unadjusted markers as well as markers adjusted with three different sets of biological variables: (a) a model consisting of age, smoking status, and triglyceride levels, (b) the SCORE, and (c) Framingham scores. We adjusted by combining the biological variables of each set into one new variable by a linear weighing with their *β*-weights derived by a Cox regression. This new variable was then included in another Cox regression model for the imaging marker we wanted to adjust. The resulting *β*-weight for the imaging marker determines the biologically adjusted prognostic power.

To analyse the complementarity of the AAC markers, a backwards stepwise deletion Cox regression model with all AAC markers was built. Least significant markers were successively deleted until only markers with significant *β*-values (*P* < 0.05) were left. This way, single markers that complemented each other and gave supplementary information were identified.

## 3. Results

**Figure 4:**
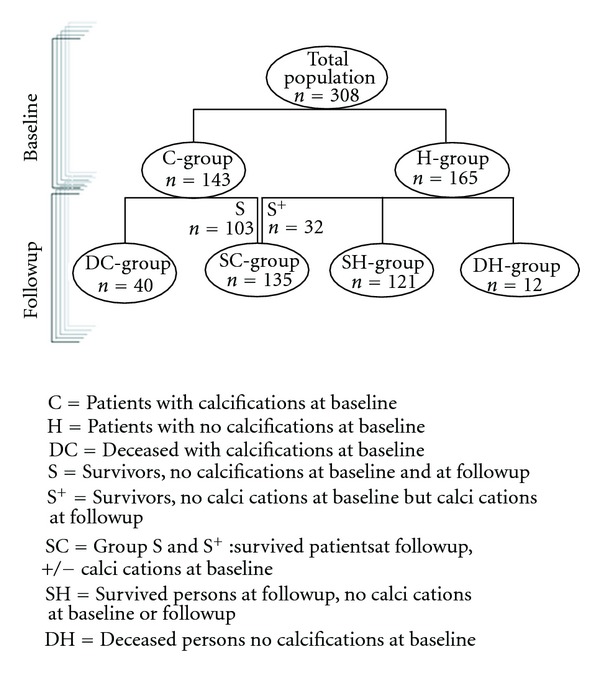
A schematic overview of the study population.

The data consisted of baseline images taken in 1992 from 308 subjects. Of these, 121 subjects had no calcifications at baseline or followup. Of the remaining 187 subjects, 52 had died before followup due to cancer (*n* = 27), CVD (*n* = 20), or other causes (*n* = 5), and 135 surviving subjects had varying degrees of abdominal aortic calcification at baseline or followup. A schematic overview of the study population is given in Figure 4, while an overview of the physical and metabolic measurements is given in Table 1.

The radiologist and computer-based AC24 scores for the 135 calcified images were in excellent agreement (Kendall's *κ* = 0.97, *P* < 0.0001).

**Figure 5:**
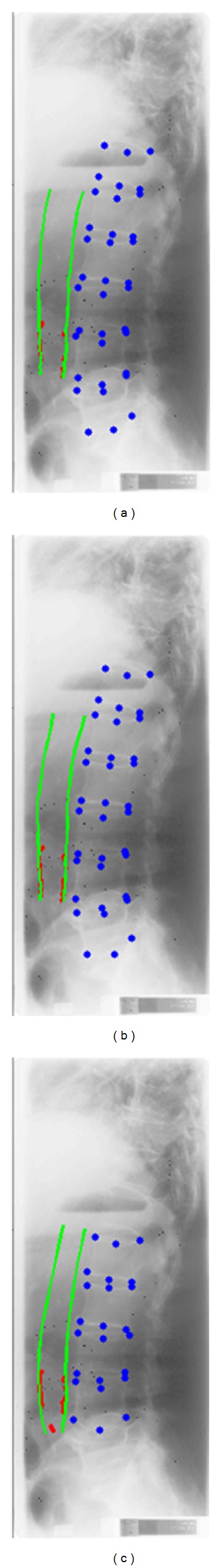
An X-ray of a participant in the EPI followup population. (a): an annotation by a radiologist. (b): a second annotation by the same radiologist. (c): an annotation done by another radiologist.

On the set of 8 images with four annotations each, the mean Jaccard Index between the radiologists' AAC outlines was 0.56 ± 0.14 (0.24–0.79) for the intraobserver variation and 0.51 ± 0.13 (0.29–0.73) for the interobserver variation, for an example, see Figure 5. The two radiologists had an intraobserver variability of 0.53 ± 0.14 (0.24–0.65) and 0.59 ± 0.14 (0.38–0.79), respectively. The CV values for the AAC marker precision on the same set of 8 images were between 12.5% and 24.9% (Table 2).

The mean values and respective standard deviations of each of the AAC markers can be found in Table 3. There was a clear difference between the means in the CVD-death and cancer-death groups compared to the survivors.

Table 4 shows that the simulated area percentage and number of calcification (NCD) have the largest individual predictive power (HR = 2.96, *P* < 0.001 and HR = 2.44, *P* < 0.001) for CVD mortality. Their hazard ratio is between 2.0 and 2.96 and 1.76 and 2.44, respectively, for the CVD-death group and between 1.68 and 2.32 and 1.69 and 2.28, respectively, for the combined CVD/cancer-death group. All hazard ratios are significantly different from unity (*P* < 0.01) both before and after adjusting for three different biological models. AC24's unadjusted individual predictive power is lower (HR = 1.66, *P* < 0.001). After adjustment for the three different biological models, the significance of the hazard ratios for AC24 is reduced and in some cases removed, leading to a hazard ratio between 0 and 1.66 for the CVD-death group and between 1.29 and 1.64 for the CVD/cancer-death group.

The results of the combined predictive power of the seven imaging markers can be seen for the CVD and the CVD/cancer group in Table 5. When combining the markers in a Cox regression model, only area percentage and NCD remained significant (*P*_area_ < 0.001, *P*_NCD_ < 0.001).

## 4. Discussion 

We evaluated whether a radiologist's manual scoring of the AC24 correlated with a computer-based scoring of the AC24 derived from a radiologist's manual outline of the calcifications on a digitized radiograph. The Kendall's coefficient of concordance showed the two scorings were in excellent agreement. Further we evaluated inter- and intraobserver variability of manual annotations using the Jaccard Index and coefficients of variation of the AAC markers, including the AC24. Although the Jaccard Index showed that the variation in the outlined calcified deposits was high, the coefficients of variation for the AC24 and the other AAC markers based on the outlines were relatively low. These results demonstrated that, even though the outlining of the individual plaques is a challenging task, the resulting markers based on the annotations provided reasonably precise measurements.

In the course of the 8-9-year-long study, 52 people died, of whom 20 died from CVD-related causes and 27 from cancer. The Cox regression models showed similar correlations to CVD and CVD/cancer mortality for the different markers. Since cancer and CVD have many overlapping pathogenetic factors, this is no surprise. The simulated area percentage and the number of calcified deposits could individually predict CVD and CVD/cancer death and contained additional information for CVD mortality even after adjustments for age, triglycerides and cholesterol, and the SCORE model and Framingham score. Hence, in this post hoc study, they predicted CVD mortality independently from traditional risk factors, in contrast to AC24. A reason for this could be that the AC24 does not discriminate between severity and spread of individual calcifications.

The risk of death due to myocardial infarct (MI) may be related to the number of active plaques [30]. During plaque development, smaller plaques develop into larger complicated lesions that either rupture or become stable plaques [31, 32]. Smaller lipid-laden plaques with high turnover have been identified as those most likely to rupture and consequent in MI [31, 32]. Thus, a large number of smaller calcifications may indicate a higher risk of rupture than few large, stable, calcifications in the same area. Techniques for measuring different aspects of plaques, such as size, distribution, and number, are in part captured by the simulated area percentage and the number of calcified deposits. This higher emphasis on the number of calcifications, rather than the total calcium burden, may reflect aspects of vulnerability that help improve the CVD-mortality prediction as observed in this work.

The Cox regression combination model showed that, when combining all the AAC markers into one model and deleting the markers that do not significantly contribute to the combined marker, only area percentage and the number of calcified deposits remained. This shows that these two AAC markers offer complementary and highly significant information about the risk of death. The complementarity of area percentage and number of calcifications suggests that size and spread of the calcifications both play important roles in atherosclerosis.

The sample size is a limitation of the present study. The relatively small population with only 20 CVD deaths, a limited representation of ethnicity and gender, and a mixture of death causes may limit the generalizability of our results. Therefore, the presented findings need to be validated in larger, independent studies. A limitation of the proposed markers could be the cost of manual annotations, but efforts have been made to automate annotations of calcified deposits [33, 34].

Compared to markers of CVD obtained with other imaging modalities, such as carotid IMT or CAC, a clear advantage of using standard radiographs is the availability of large, long duration osteoporosis screening studies [16–18]. For example, such historical data was used to verify the developed AAC markers and can improve understanding of CVD death risk factors. The clinical applicability of AAC markers can be increased if the same radiographs are used for osteoporosis screening and CVD risk assessment.

While AC24 captures essential information about AAC, the results demonstrate that some of these novel morphometric markers of AAC may capture complementary information. Therefore, the proposed radiographic AAC markers may enable improved screening for and monitoring of CVD mortality risk.

## Figures and Tables

**Table 1:** The mean and standard deviation of the measured metabolic and physical markers.

Physical/metabolic markers	Population (*n* = 308)	Survivors (*n* = 256)	Deceased (all-cause) (*n* = 52)
Age (years)	60.3 ± 7.5	59.3 ± 7.1	65.6 ± 7.0
Waist (cm)	80.7 ± 10.9	80.2 ± 9.9	83.1 ± 12.4
Waist-to-hip ratio	0.80 ± 0.08	0.80 ± 0.08	0.83 ± 0.10
Body mass index (kg/m^2^)	24.7 ± 3.9	24.7 ± 3.8	25.1 ± 4.6
Smoking (%)	37	33	58
Systolic BP (mm Hg)	127 ± 21	125 ± 20	136 ± 26
Diastolic BP (mm Hg)	77 ± 10	76 ± 10	77 ± 11
Hypertension (%)	16	15	17
Glucose (mmol/L)	5.44 ± 1.27	5.37 ± 0.99	5.79 ± 2.17
Total cholesterol (mmol/L)	6.44 ± 1.19	6.36 ± 1.14	6.85 ± 1.33
Triglycerides (mmol/L)	1.24 ± 0.75	1.15 ± 0.56	1.69 ± 1.25
LDL-C (mmol/L)	2.89 ± 0.82	2.85 ± 0.80	3.07 ± 0.93
HDL-C (mmol/L)	1.77 ± 0.48	1.77 ± 0.44	1.74 ± 0.62
ApoB/ApoA	0.57 ± 0.18	0.56 ± 0.17	0.64 ± 0.23
Lp (a) (mg/dL)	21.4 ± 21.7	21.9 ± 22.0	18.4 ± 19.8
EU SCORE	2.60 ± 2.58	2.16 ± 2.12	4.73 ± 3.45
Framingham	14.75 ± 3.54	14.21 ± 3.46	17.31 ± 2.74

**Table 2:** The inter- and intraobserver mean coefficients of variation for the AAC markers based on the inter-intra-observer test population.

Inter-intra-observer population	Interobserver CV %	Intraobserver CV %
Area %	24.1	24.9
Sim. area %	24.9	20.3
Thickness %	16.8	14
Wall %	13.0	12.5
Length %	13.0	12.5
NCD	19.4	16.6

**Table 3:** The mean ± one standard deviation of all the imaging markers stratified for the different subsets of patients. NCD^#^ stands for number of calcifications.

	All (*n* = 308)	Survivors (*n* = 256)	CVD (*n* = 20)	Cancer (*n* = 27)	CVD/Can (*n* = 47)	Other (*n* = 5)	All-cause (*n* = 52)
AC24	1.67 ± 2.55	1.35 ± 2.34	3.50 ± 2.35	3.41 ± 3.23	3.45 ± 2.86	1.35 ± 2.36	3.23 ± 2.86
Area % (%)	0.6 ± 1.2	0.5 ± 1.1	1.0 ± 0.9	1.6 ± 1.8	1.3 ± 1.5	0.5 ± 1.1	1.2 ± 1.5
Sim. area % (%)	11 ± 17	8.9 ± 15.7	24 ± 16	25 ± 24	25 ± 21	8.7 ± 15.5	23 ± 21
Thickness % (%)	11 ± 20	9.0 ± *l*9	17 ± 16	25 ± 28	21 ± 24	8.7 ± 19	20 ± 24
Wall % (%)	1.03 ± 1.83	0.79 ± 1.64	2.08 ± 1.70	2.51 ± 2.68	2.33 ± 2.30	0.80 ± 1.63	2.16 ± 2.27
Length % (%)	7.5 ± 12.8	6.0 ± 11.7	15.4 ± 11.2	17.3 ± 17.6	16.5 ± 15.1	5.9 ± 11.6	15.4 ± 15.0
NCD^#^	3.8 ± 7.7	2.6 ± 6.4	8.5 ± 6.5	11.6 ± 13.4	10.3 ± 11.0	2.6 ± 6.3	9.6 ± 10.8

**Table 4:** The relative risk per standard deviation increase in marker values stratified into death cause and adjusted for physical/metabolic markers, EU SCORE, and Framingham score, respectively. The symbols *, **, and *** denote the significance corresponding to *P* < 0.05, *P* < 0.01, and *P* < 0.001, respectively. NCD^#^ stands for number of calcifications.

	Hazard ratio not adjusted	Hazard ratio bioadjusted	Hazard ratio SCORE-adjusted	Hazard ratio Framingham-adjusted
AC24				
CVD	1.66 (1.25–2.19)***	NS	1.38 (1.02–1.86)*	NS
CVD/cancer	1.64 (1.35–2.00)***	1.31 (1.06–1.63)*	1.40 (1.13–1.72)**	1.29 (1.02–1.63)*

Area%				
CVD	1.60 (1.16–2.20)**	NS	NS	NS
CVD/cancer	1.68 (1.36–2.09)***	1.32 (1.04–1.66)*	1.47 (1.16–1.86)**	1.34 (1.04–1.72)*

Sim. area%				
CVD	2.96 (1.76–4.99)***	2.00 (1.15–3.49)*	2.46 (1.41–4.27)**	2.27 (1.26–4.09)**
CVD/cancer	2.37 (1.73–3.25)***	1.68 (1.20–2.34)**	1.96 (1.40–2.73)***	1.79 (1.26–2.54)**

Thickness%				
CVD	NS	NS	NS	NS
CVD/cancer	1.45(1.20–1.75)***	NS	1.27 (1.04–1.55)*	NS

Wall%				
CVD	1.50 (1.16–1.95)**	NS	NS	NS
CVD/cancer	1.60 (1.34–1.91)***	1.26 (1.04–1.53)*	1.42 (1.17–1.73)***	1.30 (1.05–1.62)*

Length%				
CVD	1.55 (1.18–2.04)**	NS	NS	NS
CVD/cancer	1.61 (1.34–1.95)***	1.26 (1.03–1.55)*	1.42 (1.16–1.73)***	1.29 (1.03–1.62)*

NCD^#^				
CVD	2.44 (1.72–3.48)***	1.76 (1.20–2.60)**	2.20 (1.48–3.26)***	2.04 (1.34–3.12)***
CVD/cancer	2.28(1.79–2.90)***	1.69 (1.30–2.21)***	2.00 (1.53–2.62)***	1.86 (1.40–2.47)***

**Table 5:** The individual hazard ratios for the markers in the CVD and the CVD/cancer group as well as two Cox regression elimination models. First the nonadjusted hazard ratios from Table 2 are stated again, and then two elimination models are shown. The symbols *, **, and *** denote the significance corresponding to *P* < 0.05, *P* < 0.01, and *P* < 0.001, respectively. ^#^NCD stands for number of calcifications.

	CVD: *β* · *std*	CVD Elim.: *β* · *std*	CVD/cancer: *β* · *std*	CVD/cancer Elim.: *β* · *std*
AC24	1.66***	—	1.64***	—
Area %	1.60**	−3.84***	1.68***	2.39***
Sim. area %	2.96***	—	2.37***	—
Thickness %	1.32	—	1.45***	—
Wall %	1.50**	—	1.60***	—
Length %	1.55**	—	1.61***	—
NCD^#^	2.44***	2.76***	2.28***	1.88***
